# Genomic Alterations of Tumors in HER2-Low Breast Cancers

**DOI:** 10.3390/ijms25021318

**Published:** 2024-01-21

**Authors:** Yi-Fang Tsai, Chi-Cheng Huang, Chih-Yi Hsu, Chin-Jung Feng, Yen-Shu Lin, Ta-Chung Chao, Jiun-I Lai, Pei-Ju Lien, Chun-Yu Liu, Jen-Hwey Chiu, Ling-Ming Tseng

**Affiliations:** 1 Comprehensive Breast Health Center, Taipei Veterans General Hospital, Taipei 112201, Taiwan; yftsai@vghtpe.gov.tw (Y.-F.T.); chishenh74@gmail.com (C.-C.H.); cjf0205@gmail.com (C.-J.F.); yslin@vghtpe.gov.tw (Y.-S.L.); tcchao@vghtpe.gov.tw (T.-C.C.); jilai@nycu.edu.tw (J.-I.L.); prlain@vghtpe.gov.tw (P.-J.L.); cyliu3@gmail.com (C.-Y.L.); lmtseng@vghtpe.gov.tw (L.-M.T.); 2 Division of Breast Surgery, Department of Surgery, Taipei Veterans General Hospital, Taipei 112201, Taiwan; 3 Faculty of Medicine, School of Medicine, National Yang Ming Chiao Tung University, Taipei 112201, Taiwan; cyhsu@vghtpe.gov.tw; 4 School of Public Health, College of Public Health, National Taiwan University, Taipei 112201, Taiwan; 5 Department of Pathology and Laboratory Medicine, Taipei Veterans General Hospital, Taipei 112201, Taiwan; 6 College of Nursing, National Taipei University of Nursing and Health Sciences, Taipei 112201, Taiwan; 7 Division of Plastic Surgery, Department of Surgery, Taipei Veterans General Hospital, Taipei 112201, Taiwan; 8 Division of Chemotherapy, Department of Oncology, Taipei 112201, Taiwan; 9 Division of Transfusion Medicine, Department of Medicine, Taipei Veterans General Hospital, Taipei 112201, Taiwan; 10 Division of General Surgery, Department of Surgery, Cheng-Hsin General Hospital, Taipei 112201, Taiwan

**Keywords:** breast cancer, human epidermal growth factor receptor-2, HER2-low carcinoma, next-generation sequencing

## Abstract

The aim of this study was to elucidate molecular profiling in HER2-low tumors based on a promising dataset. A total of 615 consecutive HER2-negative breast cancer samples were assayed. The genomic mutations in the two groups with different HER2 expression levels (HER2-0 vs. HER2-low) were compared. The mutation types obtained via next-generation targeted sequencing were correlated with the clinicopathological features of the patients with HER2-0 and HER2-low breast cancer. The results showed that there was a significantly higher percentage of receptor-positive (ER/PR) tumors and more low-level Ki-67 tumors, but a lower incidence of stage I/II tumors in the HER2-low group compared to the HER2-0 group. There was a significantly higher frequency of 17.62% (65/369) for *PIK3CA*_SNA in the HER2-low group than in the HER2-0 group, which had a frequency of only 9.35% (23/246) (*p* = 0.006). When the called gene alterations in the triple-negative breast cancer (TNBC) group were compared with those in the luminal-like breast cancer group, there was a significantly high frequency of 28.17% (140/497) for *ERBB2*_SNA in a luminal-like group than in the TNBC group(16.95% (20/118)).We conclude that the early detection of *PIK3CA* mutations is likely to be important and might help therapeutic decision making in patients with HER2-low tumors.

## 1. Introduction

Breast cancer is a complex disease that displays heterogeneity at the genomic, transcriptomic, and proteomic levels, as well as existing in a variety of different cellular microenvironments [1]. A simplified categorization, based on the expression level of the estrogen receptor (ER), the progesterone receptor (PR), and the human epidermal growth factor 2 (HER2), has been adopted in clinical practice, and this has allowed breast cancers to be grouped into three distinct subtypes: luminal-like tumors (ER-positive and/or PR-positive and HER2-negative), HER2-positive tumors (HER2-positive, any ER, and PR-positive), and triple-negative (ER-negative, PR-negative, and HER2-negative) tumors. The recommended strategies to treat these tumors vary distinctly on account of their different biology and responsiveness to treatment. Anti-HER2 therapies have been demonstrated to have promising efficacy against HER2-positive breast cancers; however, the benefits of such a treatment have not yet been translated to those tumors that do not have HER2 overexpression [2]. The addition of trastuzumab to chemotherapy treatment does not improve invasive disease-free survival among patients when the breast cancer does not overexpress HER2. Previous guidelines have therefore limited the application of HER2-directed therapies only toward tumors that show the overexpression and/or amplification of HER2; this is defined as a score of 3+ on immunohistochemical (IHC) analysis or *HER2* gene amplification defined via in situ hybridization (ISH) [3].

HER2-low expression, currently defined as an IHC score of 1+ or 2+ with no amplification of the *HER2* gene when analyzed via ISH assay [4,5], has currently resulted in tumors being classified as luminal-like or triple-negative breast cancers. Among patients with HER2-low tumors, progressing from the first line of therapy for metastatic breast cancers means that chemotherapy is often considered the next strategy. Breast cancer with a low expression of HER2 is a new area of interest in breast cancer research. Recent clinical trials have demonstrated significant clinical benefit when new-generation antibody–drug conjugate (ADC) treatments are implemented for metastatic breast cancer patients with HER2-low tumors [6]. These promising results have created a brand-new landscape in the era of ADC and have broken through the borders of previous subtype groupings. Here, we analyzed the clinicopathological features and explored the molecular profiling of HER2-low tumors using a previously established dataset and tried to determine if there are significant differences between HER2-0 tumors and HER2-low tumors in terms of their molecular profiles.

## 2. Results

### 2.1. A Comparison of the Clinical and Pathological Features between the HER2-0 and HER2-Low Groups

In our database, 671 patients were recorded as HER2-negative. When correlated with NGS target sequencing data, those tumor samples with a non-pass quality and a coverage of <250 were excluded. This gave a total of 615 patients with HER2-negative tumors, namely HER2-0 (IHC score 0, n = 246) and HER2-low (IHC score 1+/2+ and FISH negative, n = 369), and these were enrolled in the present study (Supplementary Information Figure S1). Those with HER2 IHC 3+ or IHC 2+/FISH+ were excluded. The clinicopathological features of this cohort study stratified by their HER2-0 and HER2-low status tumors were then analyzed. Clinical parameters, such as age, tumor size, and lymph node status, showed no statistically significant differences between HER2-0 and HER2-low tumors (Table 1).

However, notably, there was a significantly lower incidence of stage I/II samples and a significantly higher percentage of receptor (ER/PR) positive samples in the HER2-low group compared to the HER2-0 group. In contrast, there was a significantly higher percentage of tumors grade III and Ki-67 ≥ 30% in the HER2-0 group compared to the HER2-low group (Table 2) (*p* < 0.05, Chi-Square test or Fisher’s exact test).

### 2.2. A Comparison of Mutation Types between the HER2-0 and HER2-Low Groups

The Venn diagram comparison between HER2-0 and HER2-low groups is shown in Supplementary Information Figure S1. The results of the TMO assay revealed that the average number of called variants in the HER2-0 group was 4.57 (SD: 5.06, range: 1~68), while those in the HER2-low group were5.60 (SD: 13.37, range: 1~190). The mutation types of called variants were SNA, such as synonymous, missense, insertion/deletion (Indel), or frameshift, and SA, such as fusion, truncation, and CNA. The average number of each mutation type among HER2-0 and HER2-low breast cancers with at least one variant was demonstrated in Table 3.

### 2.3. Actionable Genes between HER2-0 and HER2-Low Groups

Based on ESCAT criteria, the actionable gene variants were *AKT2*, *BCRA1*, *BRCA2*, *ERBB2*, *ERBB3*, *PI3KCA*, *PTEN*, and *MDM2*. The average actionable gene variants among HER2-0 and HER2-low breast cancers are shown in Table 4.

### 2.4. Comparison of Mutational Alterations Present between the HER2-0 and HER2-Low Groups

**Figure 1:**
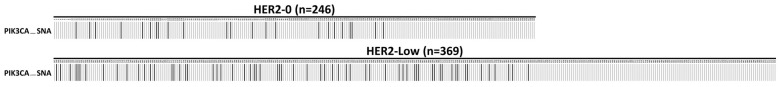
An oncoplot comparison of single nucleotide alteration (SNA) between HER2-0 and HER2-low groups. The PIK3CA_SNA is at exon 20 (the H1047R mutation). The HER2-0 is defined as IHC score 0 group (n = 246) and the HER2-low as IHC score 1+/2+ and FISH negative group (n = 369). The x-axis indicates subject number, while y-axis indicates PIK3CA_SNA mutation. The gray bars indicate no alteration compared to the reference genome, and the back bars indicate gene alteration noticed.

When the called gene alterations in the HER2-0 group were compared with those in the HER2-low group, there was a significantly higher frequency of 17.62% (65/369) for *PIK3CA*_SNA in HER2-low group than in the HER2-0 group, which had sample frequency of 9.35% (23/246) (*p* = 0.006, Chi-Square test). The oncoplot is presented in Figure 1.

**Figure 2:**
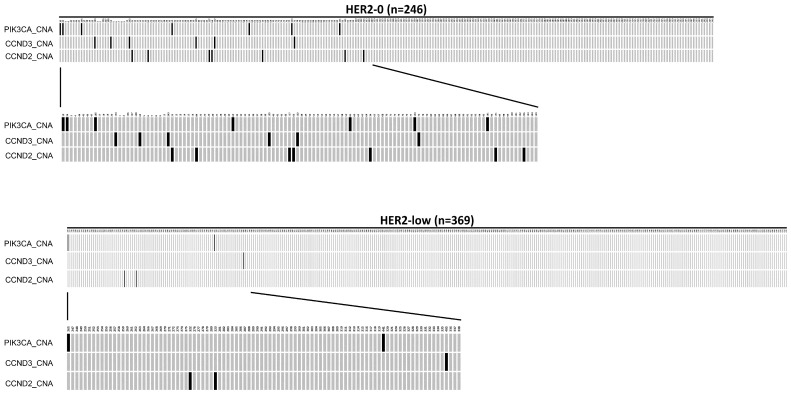
An oncoplot comparison of copy number alteration (CAN) between HER2-0 and HER2-low groups. The HER2-0 is defined as IHC score 0 group (n = 246) and the HER2-low as IHC score 1+/2+ and FISH negative group (n = 369). The x-axis indicates subject number, while y-axis indicates gene alterations such as PIK3CA_CNA, CCND2_CNA, and CCND3_CNA mutations. The gray bars indicate no alteration compared to the reference genome, and the back bars indicate gene alteration noticed.

Among the 246 HER2-0 patients, 112 (45.5%) tumors showed copy number alteration (CNA), while 185 tumors (50.1%) in the 369 HER2-low patients had CNA mutations. In addition, there was a significantly higher frequency of *PIK3CA*_CNA (2.85%), *CCND3*_CNA (2.44%), and *CCND2*_CNA (2.85%) mutations in the HER2-0 group than in the HER2-low group, which had frequencies of 0.54%, 0.27%, and 0.54%, respectively (*p* < 0.05, Chi-Square test). The oncoplot result is presented in Figure 2. The mutation details of Figure 1 and Figure 2 are shown in Table 5.

### 2.5. Comparison of the Mutational Alterations between TNBC Breast Cancer and Luminal-Like Breast Cancer in HER2-Low Tumors

**Figure 3:**
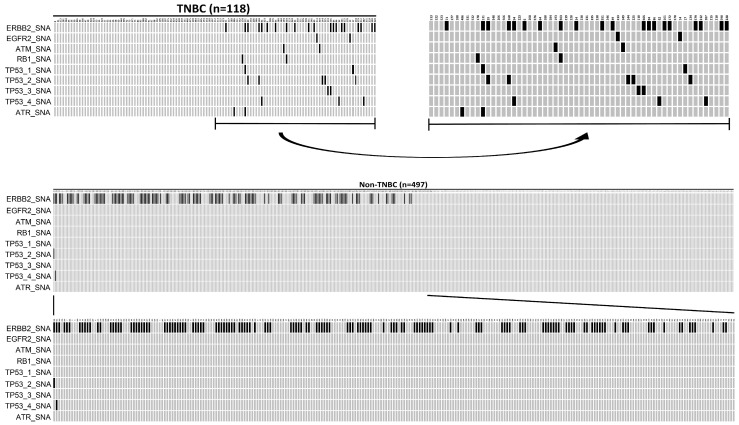
An oncoplot comparison of mutations between triple-negative breast cancer (TNBC) and Luminal-like groups. TNBC is defined as ER (-), PR (-), and HER2 (-), while luminal-like is defined as ER (+) or PR (+) and HER2 (-).The x-axis indicates subject number, while y-axis indicates gene alterations such as *ERBB2*_SNA, *EGFR2*_SNA, *TAM*_SNA, *RB1*_SNA, *TP53-1*_SNA, *TP53-2*_SNA, *TP53-3*_SNA, *TP53-4*_SNA, and *ATR*_SNA mutations. The gray bars indicate no alteration compared to the reference genome, and the back bars indicate gene alteration noticed.

In this 615-patient cohort study, 118 (19.19%) were TNBC, while 497 (80.81%) were luminal-like tumors. When the called gene alterations in the TNBC group were compared with those identified in the luminal-like group, there was a significantly high sample frequency of 28.17% (140/497) for *ERBB2*_SNA in a luminal-like group compared to the TNBC group, where the frequency was 16.95% (20/118) (*p* = 0.014, Fisher’s exact test). In contrast, there was a significantly higher sample frequency of SA (*ATR*/*ATM*) and SNA (*FGFR2*, *RB1*, *TP53*) in the TNBC group compared to the luminal-like group (*p* < 0.05, Fisher’s exact test). The oncoplot result is presented in Figure 3.The mutation details of Figure 3 are shown in Table 6.

## 3. Discussion

With the rapid advances in novel ADC regimens, it has become crucial to understand the biology and corresponding genomes of HER2-low tumors in order to define the population that can truly benefit from ADC. Here, we explored the genomic alterations in HER2-low tumors compared with HER2-0 tumors. The impressive results from the phase III DESTINY-Breast04 trial demonstrated the survival benefit of Trastuzumab deruxtecan (T-DXd) in patients with pretreated HER2-low metastatic breast cancer compared with conventional chemotherapy [6]. The revolutionary nature of ADC treatment has changed the destiny of advanced HER2-low breast cancer patients and upturned conventional treatment algorithms. Moreover, this development reshapes the way we think of the HER2-low expression phenotype, breaking through the borders between subtypes grouping and opening up discussion on how to recognize HER2-low tumors. Our work provides evidence to understand the difference in genomic mutations between the two entities, which were both defined as HER2-negative tumors before. To date, most studies of HER2-low tumors focused on the analysis of clinicopathological features and prognostic information, lacking the viewpoint from molecular profiling. Based on a previously comprehensive study, the VGH-TAYLOR trial, we elucidate the genomic alterations of HER2-low tumors from the updated 648 samples of Taiwanese breast cancer patients undergoing targeted sequencing [7,8]. Our study demonstrated that the HER2-low tumors presented more ER/PR-positive and higher percentage of low proliferative index (Ki-67 < 30%), and a significantly higher frequency for *PIK3CA*_SNA compared to HER2-0 tumors. In a previous study using large retrospective cohorts, the authors failed to find the major differences in clinicopathologic features or prognostic value between the HER2-low expression and others [9]. Another study collected retrospective PAM50 analyses to elucidate whether HER2-low and HER2-0 tumors significantly differ in gene expression, but ultimately, they found only minor differences between the two groups [4]. The study team of the DESTINY-Breast04 trial explored the potential biomarkers in collected baseline circulating tumor DNA (ctDNA) samples from 414 patients [10]. In the T-DXd and control arms, 51.3% and 54.0% of patients were found with *ESR1* mutations, and 36.1% vs. 41.6% of patients presented *PIK3CA* mutations. In patients with prior CDK4/6 inhibitors, at least one CDK4/6 inhibitor resistance marker was observed in 71.5% and 70.2% of patients. However, the efficacy of T-DXd superior to the treatment of physician choices was consistently observed independent of *ESR1*, *PIK3CA* mutation, or known resistant markers of CDK4/6 inhibitors. Whether the amazing benefit from T-DXd can be extended to the HER2-0 tumors is unknown. The minimum threshold of HER2 expression to ensure the activity of T-DXd also still needs to be determined. Although the phase II DAISY trial showed inconclusive results on their primary endpoint, objective response rate (ORR), in the HER2 non-expressing cohort [11], the ongoing larger-scale study (ClinicalTrials.gov numbers, NCT04494425) may further answer the question on HER2-0 tumors. Here, our results may help to establish the fundamental knowledge on differences between the HER2-low and HER2-0 tumors.

A previous study indicated that ER located near the cell membrane is able to activate many receptor tyrosine kinases, including epidermal growth factor receptor (EGFR) and HER2/neu (HER2) [12], and it has been suggested that this might be a possible mechanism for the presence of tamoxifen resistance in ER(+)/HER2(+) breast cancers [13]. Recently, accumulating evidence has demonstrated the cross-talk between ER and HER2 signaling is able to help identify new therapeutic strategies, including the use of aromatase inhibitors, dual blockade (trastuzumab/perstuzumab), and CDK4/6 inhibitors, to treat various different breast cancer subtypes [14,15,16,17]. The fact that there is a significantly higher percentage of receptor(ER/PR) positivity in the HER2-low group compared to the HER2-0 group might be explained by the presence of ER-HER2 signaling cross-talk in this setting.

There is accumulating evidence suggesting that the phosphatidylinositol 3-kinase (PIK3) catalytic subunit PIK3CA plays an important role in human carcinogenesis. The *PIK3CA* (H1047R) mutation has been correlated with poor clinical prognosis not only in gastric carcinoma, glioblastoma, and colorectal carcinoma [18] but also in breast cancer [19,20]. Previously, mutations affecting the *PIK3CA* gene, which results in the hyperactivation of the alpha isoform (p110a) of PI3K, have been demonstrated in 28% to 46% of patients with HR+/(HER2−) advanced breast cancers [21]. In our cohort database study, *PIK3CA* mutations were present in 38% (278/728) of all tested samples, in 43% of samples with the HR-/HER2+ subtype and in 42% of samples with HR+/HER2-post-menopausal status. Notably, when patients were treated with CDK4/6 inhibitors, the median time to treatment failure was 12 months (95% CI: 7–21 months) in the *PIK3CA* mutation group, but this increased to 16 months (95% CI: 11–23 months) in the *PIK3CA* wild-type group [22]. Thus, the *PIK3CA* mutations were associated with a reduced sensitivity to CDK4/6 inhibitor, and this is compatible with earlier findings [23,24].

There is consensus that *PIK3CA* gene mutation often results in hyperactivation of the PI3K-rapamycin (mTOR) pathway. The results obtained from the BOLERO-1/-3 study suggest that patients having tumors with a *PIK3CA* mutation or a hyperactive PI3K pathway derive PFS benefit from treatment with everolimus compared to those without such a mutation [25]. The most common mutations of *PIK3CA* in Taiwanese female breast cancers are in exon 20 (the H1047R mutation), in exon 9 (the E545K mutation), and in exon 9 (the E542K mutation) with frequencies of 41.6%, 18.9%, and 10.3%, respectively [22]. Our present results show that there is a significantly higher frequency of 17.62% (65/369) of *PIK3CA*_SNA in the HER2-low group compared to the HER2-0 group, which has a frequency of 9.35% (23/246) (*p* = 0.006, Chi-Square test) (Table 4). This suggests that early detection of *PIK3CA* mutations is important and might help therapeutic decision making in those patients with a HER2-low tumor. Although there was a higher frequency of *PIK3CA*_CNA (2.85%) in the HER2-0 group than in the HER2-low group, which has a frequency of 0.54%, the clinical significance of this remains to be elucidated due to the limited sample size.

TNBC (ER-, PR-, and HER2-) tumors are characterized by their clinicopathological features, such as their occurrence in younger women, their aggressive nature (a higher tumor grade and a higher Ki67 percentage), and a higher association with metastasis to distant organs. In patients with HER2-low tumors, it is expected that TNBC should be included. In the present study, our findings show that there is a significantly higher percentage of tumors grade III and Ki-67 ≥ 30% among the HER2-0 group compared to the HER2-low group (Table 2) (*p* < 0.05, Fisher’s exact test), which is compatible with our findings that there is a higher percentage (29.7%) of TNBC in the HER2-0 group compared to 12.2% in the HER2-low group (*p* < 0.0001, Fisher’s exact test). Based on our NGS analysis, there is also a significantly higher frequency of 28.17% (140/497) for *ERBB2*_SNA in the luminal-like group compared to the TNBC group, which has a frequency of 16.95% (20/118) (*p* = 0.014, Fisher’s exact test) (Table 6). Although previous studies have demonstrated that G to A mutation at amino acid codon 655 of the human *erbB-2/HER2* gene is a new allele polymorphism of the *ERBB2* gene [26,27], the exact role of such mutation as a therapeutic target in luminal-like breast cancer remains further clinical elucidation.

In summary, HER2-negative breast cancer is able to be further divided into the HER2-0 and HER2-low groups. There are different clinical manifestations between these two groups. There is a significantly higher frequency of 17.62% (65/369) of *PIK3CA*_SNA in the HER2-low group compared to the HER2-0, with a frequency of 9.35% (23/246). When the called gene alterations are compared between the TNBC and luminal-like groups, there is a significantly high frequency of 28.17% (140/497) for *ERBB2*_SNA in the luminal-like group compared to the TNBC group, which has a frequency of 16.95% (20/118).

## 4. Materials and Methods

### 4.1. Study Population

Under the approval of the Institutional Review Board of Taipei Veterans General Hospital (# 2023-06-025BC), we followed the same protocol as the VGH-TAYLOR, which involved a comprehensive precision medicine investigation of the heterogeneity of Taiwanese breast cancer patients (Clinical trial registration: NCT04626440 (ClinicalTrials.gov)) [28]. In short, the study comprised a broad clinical spectrum of breast cancers, namely, Group 1, those planned to receive first-line surgery followed by adjuvant therapy or having early relapse within 3 years; Group 2, those planned to receive first-line neoadjuvant therapy followed by surgery; and Group 3, those planned to receive treatment for de novo stage IV or stage IV disease with recurrence beyond 3 years. Three years (Jan. 2018–Jan. 2020) of enrollment and four years of follow-up after enrollment were included. All patients received treatment following the contemporary practice guidelines of the Comprehensive Breast Health Center at Taipei Veterans General Hospital, which is based on the NCCN and St. Gallen guidelines.

### 4.2. Pathology Review

IHC staining to detect ER (clone 6F11; Leica Biosystems, Newcastle, UK; 1:100), PR (clone 16; Leica Biosystems, Newcastle, UK; 1:150), and HER2 (Ventana PATHWAY anti-HER2/neu 4B5 rabbit monoclonal antibody), were evaluated by experienced pathologists from our institute. The positivity of ER and PR was defined as ≥1% of tumor cells exhibiting nuclear staining. HER2 IHC positivity (score 3+) was defined via complete intense membrane staining in >10% of tumor cells. Reflex in situ hybridization (ISH) testing via fluorescence ISH (PathVysion HER2 DNA Probe Kit; Abbott Laboratories, Des Plaines, IL, USA) was performed for cases that gave equivocal HER2 IHC results (score 2+). Patients with an average HER2 copy number of ≥6 signals/cell, or ≥4 signals/cell and a HER2 ISH ratio (HER2 gene signals to chromosome 17 centromere signals) of ≥2 were regarded as ISH-positive by the 2018 American Society of Clinical Oncology (ASCO)/College of American Pathologists (CAP) guidelines. In the present study, HER2-negative patients were further categorized into two subgroups: the HER2-0 (IHC score 0) group and the HER2-low (IHC score 1+/2+ and FISH-negative) groups.

### 4.3. Sample Preparation

Sample preparation was carried out to allow next-generation sequencing (NGS) targeted sequencing of the fresh-frozen paraffin-embedded (FFPE) samples. The preparation of the FFPE section was performed at the clinical site following the standard procedures. The hematoxylin and eosin (H&E) staining was performed and interpreted under the guidance of a certified pathologist (Dr. Hsu, CY). Approximately 7 unstained sections of tumor FFPE tissues per subject were retrieved; at least one unstained section was prepared for H&E staining, and 6 unstained sections were prepared for TMO comprehensive assay.

### 4.4. DNA Extraction

The extraction of DNA was conducted in the central laboratory according to the appropriate laboratory manuals. In short, DNA extraction from FFPE or tissue cores wassubjected to xylene treatment and rehydrated using a series of ethanol washes, followed by the removal of proteins (nucleases) via proteinase K digestion. Nucleic acids were purified from the tissue lysate, and this was followed by phenol extractions, and RNase A was added to eliminate RNA contamination. The addition of sodium acetate and isopropanol precipitated DNA, and high-speed centrifugation was used to pellet the DNA, followed by the salt out process and washing with 70% ethanol and by centrifugation to re-pellet the DNA and stored at −20 °C for further use. Purified DNA can subsequently be used in downstream applications, which include PCR, array comparative genomic hybridization 4 (array CGH), methylated DNA Immunoprecipitation (MeDIP), and sequencing, allowing for an integrative analysis of tissue/tumor samples. Additional sections of FFPE samples of individual subjects might be pursued if a sample failed the nucleic acid quality check. The criteria for the DNA quality check followed the manual of the TMO assay requirement (see below).

### 4.5. Oncomine^TM^ Comprehensive Assay (TMO Comprehensive Assay)

Targeted NGS experiments were performed using the TMO comprehensive assayv3 from FFPE tissues (Thermo Fisher Scientific, Waltham, MA, USA) in order to detect thousands of variants across 161 genes that are relevant to cancer. The TMO comprehensive assay was performed using the collected FFPE tissue samples, and the analyses of the TMO comprehensive assays included a range of specific genes and various types of mutation within these genes, including frameshift, missense, synonymous, single nucleotide alteration (SNA), insertion/deletion (Indel), structure alteration (SA) and copy number alteration (CNA); each analysis was for an individual subject.

**Figure 4:**
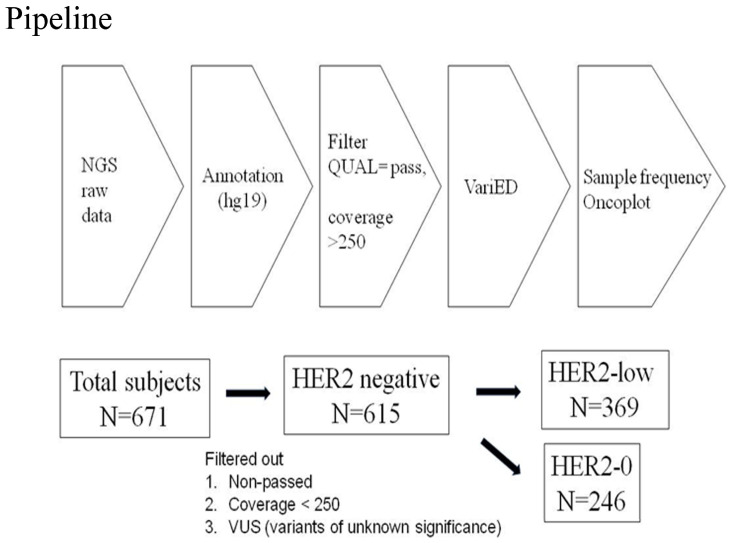
Pipeline of next-generation sequencing analysis in this study. From 2018-1 to 2020-1, a total of 671 patients with HER2-negative tumors were included. The raw data of NGS were annotated usinghg19 human genome reference and VariED database. The statistical significance of sample frequency of mutations was selected between HER2-0 andHER2-low groups with a *p* value < 0.05 via Chi-square test or Fisher’s exact test. Among 671 tumors receiving NGS target sequencing, those data with non-pass quality, coverage < 250, and variants with unknown significance were excluded. Finally, 615 patients with HER2-negative tumors were categorized into HER2-0 (n = 246) and HER2-low (n = 369) groups. The definition of HER2-0 and HER2-low was HER2-0, immunohistochemistry score (IHC) 0, and HER2-low, IHC score 1+/2+, and FISH-negative, respectively.

Amplicon libraries were constructed using multiplex PCR primers after the preparation of DNA from the FFPE samples. Sequencing was performed using Ion Gene Studio S5 System and Ion 540 Chips. Raw data processing, alignment, and variant calling were performed using v3–w3.2–DNA and Fusions–Single Sample version 5.10, with the variant calling by the Torrent Variant Caller plug-in. Further management involved Ion Reporter^TM^ Software with the workflow “Oncomine Comprehensive v3–w3.2–DNA and Fusions–Single Sample” version 5.10 being selected and the filter chain “Oncomine Variants” version 5.10 being applied [7]. The pipeline of next-generation sequencing analysis in this study is described in Figure 4.

### 4.6. Statistical Methods

Descriptive statistical analysis was applied to compare baseline characteristics among the risk assessment and clinicopathological features. Categorical data were summarized in counts and percentages. The Chi-Square test or Fisher’s exact test was used to compare the distributions of categorical variables. A two-sided *p*-value of <0.05 was considered statistically significant. All statistical analyses were performed using Python software (version 3.9.6. Python Software Foundation, Wilmington, Del; open source; https://www.python.org/ (accessed on 8 July 2023))

## 5. Conclusions

We conclude that there are different clinical manifestations present in the HER2-0 group and the HER2-low group. There is also a significantly higher frequency of *PIK3CA*_SNA in the HER2-low group than in the HER2-0 group. It is suggested that the early detection of the presence of a *PIK3CA* mutation is important and might help therapeutic decision making in patients with HER2-low tumors.

## Figures and Tables

**Table 1:** Clinical presentations by different immunohistochemical scores in HER2-negativebreast cancer.

	HER2-0 (n = 246)	HER2-Low (n = 369)	*p* Value *
Age (years)			0.742
<55	120 (48.7%)	175 (47.4%)	
≥55	126 (51.2%)	194 (52.5%)	
Tumor size			0.878
T1 (≤2cm)	87 (35.3%)	146 (39.5%)	
T2 (2.1–5 cm)	126(51.2%)	166 (44.9%)	
T3 (>5 cm)	20 (8.1%)	24(6.5%)	
T4	12 (4.8%)	33(8.9%)	
NA	1 (0.4%)	0 (0%)	
Node			0.182
N0	131 (53.2%)	196 (53.1%)	
N1	74 (30.1%)	90 (24.4%)	
N2	25 (10.2%)	42 (11.4%)	
N3	14 (5.7%)	39 (10.6%)	
NA	2 (0.8%)	2 (0.5%)	
Stage			0.020 *
I	68 (27.7%)	116 (31.4%)	
II	121 (49.2%)	137 (37.1%)	
III	35 (14.2%)	77 (20.9%)	
IV	22 (8.9%)	39 (10.6%)	

HER2-0, HER2 immunohistochemistry score = 0; HER2-low was defined as HER2 immunohistochemistry score (+/++ with FISH (-); * *p* value via Chi-Square or Fisher’s exact test.

**Table 2:** Pathological features by different immunohistochemical scores in HER-2 negative breast cancer.

	HER2-0 (n = 246)	HER2-Low (n = 369)	*p* Value *
Grade			0.0001 *
I	32 (13.0%)	64 (17.3%)	
II	119 (48.4%)	234 (63.4%)	
III NA	86 (35.0%) 9 (3.6%)	67 (18.2%) 4 (1.1%)	
ER			0.0001 *
Negative (<1%)	73 (29.7%)	46 (12.5%)	
Positive (≥1%)	173 (70.3%)	323 (87.5%)	
PR			0.0001 *
Negative (<1%)	90 (36.6%)	83 (22.5%)	
Positive (≥1%)	156 (63.4%)	286 (77.5%)	
Ki-67			0.0001 *
<30%	120 (48.8%)	249 (67.5%)	
≥30%	120 (48.8%)	117 (31.7%)	
NA	6 (2.4%)	3 (0.8%)	

ER, estrogen receptor; PR, progesterone receptor; NA, not available; HER2-0, HER2 immunohistochemistry score = 0; HER2-low was defined as HER2 immunohistochemistry score (+/++ with FISH (-); * *p* value via Chi-Square or Fisher exact test.

**Table 3:** Comparisons of mutation types between HER2-0 and HER2-low tumors.

Subtype	Mutation Type	Mean	SD	Min	Max
**HER2-0** (246/1124) *	CNA	1.252	1.872	0	9
(Mean: 4.57	Fusion	0.098	0.311	0	2
SD: 5.06	Frameshift Deletion	0.207	0.463	0	2
Min: 1	Frameshift Insertion	0.378	1.738	0	24
Max: 68)	Missense	2.110	1.414	0	17
	Non-frameshift Deletion	0.016	0.127	0	1
	Nonsense	0.508	3.287	0	51
**HER2-low** (369/2086) *	CNA	1.238	1.896	0	11
(Mean: 5.60	Fusion	0.122	0.344	0	2
SD: 13.37	Frameshift Deletion	0.192	0.428	0	3
Min: 1	Frameshift Insertion	1.141	10.326	0	174
Max: 190)	Missense	2.247	1.887	0	26
	Non-frameshift Deletion	0.024	0.154	0	1
	Nonsense	0.640	5.888	0	96

* Subtype (subject number/called variant number); HER2-0 and HER2-low are defined in the Section 4.

**Table 4:** Comparisons of actionable genes between HER2-0 and HER2-low tumors.

Subtype	Gene	Mean	SD	Min	Max
**HER2-0** (246) *	*AKT2*	0.012	0.110	0	1
	*BRCA1*	0.049	0.358	0	5
	*BRCA2*	0.098	0.412	0	4
	*ERBB2*	0.280	0.459	0	2
	*ERBB3*	0.004	0.064	0	1
	*ESR1*	0.008	0.128	0	2
	*PIK3CA*	0.358	0.544	0	2
	*PTEN*	0.008	0.128	0	2
	*MDM2*	0.037	0.188	0	1
**HER2-low** (369) *	*AKT2*	0.014	0.116	0	1
	*BRCA1*	0.073	0.649	0	9
	*BRCA2*	0.171	1.154	0	14
	*ERBB2*	0.317	0.494	0	3
	*ERBB3*	0.003	0.052	0	1
	*ESR1*	0.008	0.090	0	1
	*PIK3CA*	0.493	0.595	0	3
	*PTEN*	0.008	0.090	0	1
	*MDM2*	0.038	0.191	0	1

* Subtype(subject number); HER2-0 and HER2-low are defined in the Section 4.

**Table 5:** Comparison of mutation alterations between HER2-0 and HER 2-low expression tumors.

Chrom.	Region	ALT	Type	Gene	Variant ID	Type	Function	Site	Clin Var	HER2-0 (n = 246)	HER2-Low (n = 369)	*p*-Value
3	178952085	c.3140A>G	SNA	*PIK3CA*	COSM775	missense	gain	exonic	pathogenic	9.35% *	17.62% *	0.006
3	178916549	AMP	CNA	*PIK3CA*		amplification	gain	exonic		2.85%	0.54%	0.034
6	41903566	AMP	CNA	*CCND3*		amplification	gain	exonic		2.44%	0.27%	0.018
12	4383096	AMP	CNA	*CCND2*		amplification	gain	exonic		2.85%	0.54%	0.034

ALT, alteration; AMP, amplification; Chrom, chromosome; CNA, copy number alteration; SNA, single nucleotide alteration; The definition of HER2-0 and HER2-low was described in Section 4. *p* value was determined via Chi-Square test or Fisher’s exact test. Asterisk indicates sample frequency in percentage.

**Table 6:** Comparison of mutation alterations between triple-negative tumors and luminal-like tumors in HER 2-negative breast cancer.

Chrom.	Region	ALT	Type	Gene	AA	Type	Function	ClinVar	TNBC (n = 118)	Luminal-Like (n = 497)	*p*-Value
17	37879588	c.1963A>G	SNA	*ERBB2*	p.(I655V)	missense			16.95% *	28.17% *	0.014
10	123247516	c.1975A>G	SNA	*FGFR2*	p.(K659E)	missense	Gain	Likely pathogenic	1.69%	0%	0.037
11	108153584	c.3725delC	SA	*ATM*	p.(T1242fs)	frameshift Dele	Loss		1.69%	0%	0.037
13	48953760	c.1363C>T	SNA	*RB1*	p.(R455*)	nonsense	Loss	Pathogenic/Likely pa	1.69%	0%	0.037
17	7576852	c.993+1G>A	SNA	*TP53*		unknown			1.69%	0%	0.037
17	7577120	c.818G>A	SNA	*TP53*	p.(R273H)	missense	Loss	Pathogenic/Likely pa	4.24%	0.20%	0.001
17	7577539	c.742C>T	SNA	*TP53*	p.(R248W)	missense	Loss	Pathogenic/Likely pa	1.69%	0%	0.037
17	7578263	c.586C>T	SNA	*TP53*	p.(R196*)	nonsense	Loss	Pathogenic	2.54%	0.20%	0.024
3	142231209	c.4743_4744del	SA	*ATR*	p.(F1582fs)	frameshift Dele	Loss	unknown	1.69%	0%	0.037

AA, amino acid change; ALT, alteration; Chrom, chromosome; SA, structure alteration; SNA, single nucleotide alteration; TNBC, triple-negative breast cancer; The definitions of TNBC and luminal-like (non-TNBC) are described in the Section 4. *p* value was determined via Fisher’s exact test. Asterisk indicates sample frequency in percentage.

## Data Availability

All data generated or analyzed during this study are included in this published article (and its Supplementary Information files).

## Supplementary Materials

The supporting information can be downloaded at https://www.mdpi.com/article/10.3390/ijms25021318/s1.

## Abbreviations

ADC	antibody–drug conjugate
CNA	copy number alteration
ER	estrogen receptor
HER2	human epidermal growth factor receptor 2
IHC	immunohistochemistry
Indel	insertion/deletion
ISH	in situ hybridization
NGS	next-generation sequencing
pCR	pathological complete response
PR	progesterone receptor
SA	structure alteration
SNA	single nucleotide alteration
T-DXd	Trastuzumab deruxtecan
VUS	variants of unknown significance
