# Supplementary material for: Genomic Alterations of Tumors in HER2-Low Breast Cancers
Source: Int J Mol Sci. 2024 Jan 21;25(2):1318. doi: 10.3390/ijms25021318 (PMC10816179; doi:10.3390/ijms25021318)

Supplementary information Figure S1.The venn diagram of called variants and actionable genesbetween HER2-0 and HER2-low groups.(A) The number of called variant classes. (B) The number of called variants. (C) The number of actionable gene classes. (D) The number of actionable genes.

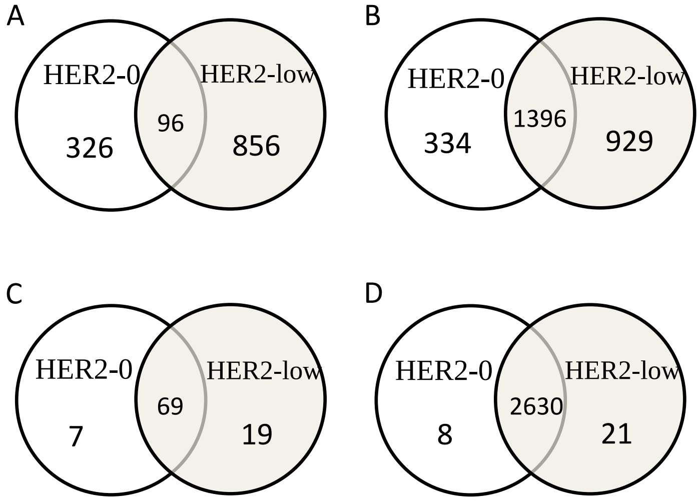

Supplement: Supplementary file 1 [file ijms-25-01318-s001.zip › ijms-2759853-supplementary.pdf]
